# Nanoscale Prussian Blue and Its Analogues: Design and Applications in Infection Control, Wound Healing and Beyond

**DOI:** 10.3390/pharmaceutics16121616

**Published:** 2024-12-20

**Authors:** Nayanika Chakraborty, Indrajit Roy, Pradeep Kumar, Swati Singh, Rajiv Pathak, Vibhav Gautam, Hemant K. Gautam

**Affiliations:** 1 Department of Chemistry, University of Delhi, Delhi 110007, India; nayanika493@gmail.com (N.C.); iroy@chemistry.du.ac.in (I.R.); 2 Department of Immunology and Infectious Disease Biology, CSIR-Institute of Genomics and Integrative Biology, Sukhdev Vihar, New Delhi 110025, India; pkumar@igib.res.in; 3 Centre of Experimental Medicine and Surgery, Institute of Medical Sciences, Banaras Hindu University, Varanasi 221005, India; swati.7672@bhu.ac.in; 4 Department of Genetics, Albert Einstein College of Medicine, Bronx, New York, NY 10461, USA; rajiv.pathak@einsteinmed.edu

**Keywords:** Prussian blue nanoparticles, biocompatibility, antibacterial agents, photothermal effects, anti-inflammatory, wound healing

## Abstract

Prussian blue nanoparticles (PBNPs) have gained significant attraction in the field of nanomedicine due to their excellent biocompatibility, potential for nanoscale production, exceptional photothermal conversion ability, and multi-enzyme mimicking capabilities. PBNPs have made considerable advancements in their application to biomedical fields. This review embarks with a comprehensive understanding of the physicochemical properties and chemical profiling of PB-based nanoparticles, discussing systematic approaches to tune their dimensions, shapes, and sizes, as well as their biomedical properties. Subsequently, the use of PB-based NPs in the biomedical sector is extensively discussed and categorized based on the various features of modified PBNPs, either in combination with drugs or their analogues. Finally, the article highlights the existing challenges associated with current studies and explores the latest developments in these rapidly evolving PB-based nanoplatforms and their therapeutic potentials. Overall, this review aims to deepen the understanding of PB-based NPs and provide crucial insights into their rational design in disease treatment.

## 1. Introduction

Prussian blue nanoparticles (PBNPs) exhibit unique characteristics as a coordination polymer and have been significantly researched in diverse sectors, including catalysis [1], ion batteries [2], spectroscopy [3], chemical sensing [4], and more recently, nanomedicine [5]. Prussian blue (PB) was accidentally discovered by the Berlin artist Diesbach in 1704 while attempting to prepare a red pigment, Florentine lake [6]. In 1936, Keggin and Miles conducted a structural analysis of PB using powder diffraction to investigate its crystal structure, which was later supplemented by single crystal diffraction analysis by Herren in 1980 [7,8]. In 2003, the US Food and Drug Administration approved PB, marketed as Radiogardase^®^, as an antidote for internal radioactive contamination with thallium and cesium-137 due to its exceptional adsorption capabilities for heavy metal ions, ion-exchange properties, and mechanical trapping abilities [9].

The crystalline framework structure of PBNPs predominantly consists of ferric ions (Fe^3+^), ferrous ions (Fe^2+^), and bridging cyanogroups. In this framework, Fe^3+^ ions are coordinately bound to the nitrogen atoms of the cyanide groups, while Fe^2+^ ions are coordinately bound to the carbon atoms of the cyanide groups. This arrangement forms a face-centred cubic (fcc) unit cell with a lattice parameter of approximately 10.2 Å, resulting in a three-dimensional coordination network, and the space group of this structure is commonly referred to as *Fm3m* [4]. Due to the characteristic Fe(II)-CN-Fe(III) linkage, PBNPs exhibit an absorption peak at 700 nm and can efficiently convert red and near-infrared (NIR) light into heat. This is attributed to the intervalence charge transfer, where an electron can migrate from Fe^2+^ to Fe^3+^ ions. The classical preparation of PB involves either a simple mixing of aqueous Fe(III) and [Fe(CN)_6_]^4−^ or an alternative preparation involving the mixing of Fe(II) salts with [Fe(CN)_6_]^3−^. PB is believed to exist in two forms: “soluble”, KFe^III^[Fe^II^(CN)_6_]·xH_2_O (where x = 1–5), and “insoluble”, Fe^III^_4_[Fe^II^(CN)_6_]_3_·xH_2_O (where x = 14–16). The differences in solubility are attributed to variations in crystal size [10]. In “insoluble” PB, water molecules fill the gaps within the defective lattice since [Fe(CN)_6_]^4−^ anions are sub-stoichiometric. These water molecules can either coordinate with iron (Fe) ions to balance the charge, indicative of a coordinative type, or enter the interior cavities and remain uncoordinated with Fe ions, indicative of a zeolite-type framework. To maintain electroneutrality in the cavities of “soluble” PB, alkali metal ions (K^+^) replace water molecules. Both aforementioned synthetic methodologies lead to the synthesis of “insoluble” PB, with the primary difference being the size of the particles, as PB can precipitate in either bulk or nanometric forms. In the past two decades, numerous papers have reported the synthesis of PB with controlled particle size and shape, highlighting the growing interest in PB nanomaterials [11].

Prussian White (K_2_Fe^II^Fe^II^(CN)_6_), Prussian Yellow (Fe^III^Fe^III^(CN)_6_), and Berlin Green (K_1/3_Fe^3+^(Fe^III^(CN)_6_)_2/3_(Fe^II^(CN)_6_)_1/3_) are the names given to the additional oxidative states of PB, each exhibiting different hues by adjusting external potentials [12]. Prussian White has no distinct absorption band, whereas PB and Berlin Green feature two absorption bands in the visible spectrum at 700 nm and 480 nm, respectively. Prussian Yellow has a single absorption band at 420 nm. In addition, the various oxidative states of PBNPs can interconvert through catalytic processes, which are the fundamental mechanisms behind the enzyme-mimicking activities and are also utilized in biosensors at low concentrations.

Despite these excellent properties, PB has recently been predominantly used in battery electrodes and biosensors rather than in disease treatment. The significant research interest in PBNPs or PB-related nanodrugs is due to several factors: (*i*) the extraordinary photothermal effect that permits the removal of bacteria and malignant cells upon exposure to near-infrared (NIR) light [13]; (*ii*) the presence of mesopores or other hollow structures in PBNPs, which can facilitate drug and enzyme delivery, thereby addressing the limitations of conventional drugs such as systemic toxicity, improper target specificity, and poor solubility [14]; and (*iii*) the enzyme-mimicking capabilities of PBNPs that can be employed to scavenge reactive oxygen species (ROS) in inflammatory diseases and enhance oxygen delivery in photodynamic treatment [15]. Additionally, the inherent bioactivities of PB-based NPs can be utilized to monitor the therapeutic efficacy and guide subsequent treatments.

This review focuses on recent developments in synthetic strategies for PBNPs (Table 1) and examines the structurally controlled functionality of these high-quality, fine-tuned nanoparticles (NPs). Additionally, we systematically discuss various applications of PBNPs, including their use as antibacterial agents (Table 2), anti-inflammatory agents, and treatments for infectious wounds (Table 3). Finally, we address the current limitations in existing studies and explore the latest developments and potential applications of PB-based nanoplatforms. In conclusion, this review seeks to establish a groundwork for future research endeavours and the development of PBNPs as a reliable therapeutic approach for various biomedical applications.

## 2. Synthetic Strategies and Structural Refinement of PBNPs

The advancement of nanomaterials and the development of synthetic strategies of the preparation methods for PBNPs have established a basis for their improved applicability, enhanced performance, and rapid clinical translation. A number of techniques have been used for the rationalization of PBNPs with controlled shape and size including the hydrothermal approach, the simple solution method, the single and double precursor method, the reversed-phase microemulsion method, and the hydrolysis method [10,45].

### 2.1. Co-Precipitation Method

The fabrication of PBNPs in the double-precursor synthesis method due to co-precipitation involves mixing of equimolar concentrations of Fe^3+^/Fe^2+^ and [Fe(CN)_6_]^4^^−^/[Fe(CN)_6_]^3^^−^ solutions. This approach offers advantages such as rapid synthesis without the need for a reducing agent. However, improvements are still needed in dispersibility, stability, and morphological control [46]. In 1998, Yang et al. presented the single precursor synthesis technique [47]. Typically, the ferricyanide complex of K_4_[Fe(CN)_6_] ions is utilized as the single precursor followed by a gradual reduction and oxidation to produce Fe in the other oxidation state. These generated Fe^3+^/Fe^2+^ ions instantly react with the precursor to produce PBNPs. The single precursor synthesis has a longer reaction time than the two-precursor synthesis and a trace of hydrogen cyanide is produced in the reaction process that may hinder the application of this method in wide-scale production, but it has greater morphological control and NP homogeneity.

Uemura and Kitagawa have combined equimolar solutions of FeCl_2_ and K_3_Fe(CN)_6_ and polyvinylpyrrolidone (PVP) in an acid solution to prepare PBNPs within a specific size range [48]. K_3_Fe(CN)_6_ releases Fe^3+^ in acid solution, sequentially Fe^3+^ ions are reduced by PVP and which, on interaction with the remaining K_3_Fe(CN)_6_, generate PBNPs with the average diameter adjusted between 12 and 27 nm while depending on the Fe ion concentration and feed ratios of Fe ion to PVP. Generally, PVP acts as a reductant and surfactant limiting the growth of the NPs. It is remarkable that throughout the course of a month, the PVP-protected PBNPs successfully maintained their stability as clusters in CHCl_3_ without additional agglomerations or dissociation, in addition to the re-dissolution into a variety of organic solvents attributed to the amphiphilic nature of PVP. The PVP-coated PBNPs absorption spectrum maxima varied with solvent polarity from 672 nm in CHCl_3_ to 689 nm in water, despite the enormous difference in dielectric constant between water (80.4 at 20 °C) and CHCl_3_ (4.8 at 20 °C). Synthetic protocols of PBNPs have diameters that are greater than 50 nm, which has an impact on the biodistribution, phagocytosis of cells, and in vivo circulation of PBNPs, thus, it becomes essential to change the growth rate of PBNPs. Shou et al. synthesized ultrasmall PBNPs via zinc doping [49]. After 10% zinc doping, the diameter of PBNPs was found to decrease from 38.5 nm to 3.8 nm. Hu et al. changed the PVP concentration to alter the particle size and shape of PBNPs. They found that at higher PVP concentrations, larger and smoother cubic PBNPs were produced, whereas smaller PBNPs with rougher surfaces were produced at lower concentrations of PVP. However, research is going on to establish the association between the therapeutic effectiveness of PBNPs and surface roughness [17].

Other surfactants including polysaccharides and polyethylene glycol (PEG) [50] have also been applied to replace PVP to prepare PBNPs. Previous research has employed a number of biocompatible polymers as protective agents, including chitosan (CS) [51], polyethylenimine (PEI) [52], oxalic acid [53], and poly (diallyldimethylammonium chloride) (PDDA) [54]. The polymeric protectors have the ability to reduce surface energy, prevent agglomeration, boost NP solubility, and so forth, while the surface microenvironment leads to tunable surface properties of the nanomaterials which have an impact on PBNPs inherent characteristics [55]. It is possible through layer-by-layer (LbL) sequential deposition to generate multilayer films made up of PB and poly(allylamine hydrochloride) (PAH) [56]. This methodology proceeded without the use of any stabilizing polymers by simply combining FeCl_3_ and K_3_Fe(CN)_6_ in the presence of a slight excess of H_2_O_2_ to produce an ampere metric transducer for biosensors based on oxidase enzymes. This synthetic method helps in the production of spherical nanostructures, measuring 5 nm instead of 50 nm using a sonochemistry-based approach. Xiaoyan He and colleagues suggested ultrasonic single-source precursor K_4_[Fe(CN)_6_] directly dissociating in acidic solution using ultrasound to produce regular, single-crystalline nanocubes of PB, Fe_4_[Fe(CN)_6_]_3_, for wide-scale applications. The reaction temperature, the concentration of K_4_[Fe(CN)_6_] aqueous solution, and the ultrasonic condition all have a significant impact on the size and size distribution of the cubes [57].

The citrate/citric acid capped synthesis is the most investigated method for synthesizing PBNPs in water without the use of any polymeric or polyelectrolyte capping agent to restrict particle growth. Initially, it was thought that citric acid could only serve as a reductant and to avoid cross-linking among gelatin molecules to coagulate by Fe(CN)_6_^3−^ ions; therefore, the usage of a protective polymeric agent (such as gelatin) was kept during the synthesis [50]. Nowadays, only Fe^III^ salt, ferrocyanide, and citric acid are being used instead of a polymer matrix or a protective cage. The citric acid produces a single-crystal-like cubic structure with a large size distribution. These particles have intriguing properties like facile surface functionalization, low cytotoxicity and high solubility in water, alcohol, and water-DMSO mixtures. Hence, citrate capping appears to be ideal for producing high-quality, shape-controlled NPs.

### 2.2. Microemulsion Method

Vaucher and colleagues were the first to report a rationalized nanometric PB production in water droplets generated by the reverse microemulsion from the anionic surfactant AOT (sodium bis(2-ethylhexyl)sulfosuccinate) in isooctane [11]. By using a gradual photoreduction of [Fe^III^(C_2_O_4_)_3_]^2−^ into Fe^2+^ in the presence of [Fe^III^(CN)_6_]^3−^ ions, the photochemical synthesis produced cubic NPs with an average size of ~12–16 nm. This photochemical synthesis resulted in hydrophobic PBNPs 2D superlattices because the head of AOT sulphate attached to the surface of these PBNPs. In another similar microemulsion technique with compressed CO_2_ as the antisolvent, separate isooctane/AOT microemulsions containing FeCl_2_ and K_3_Fe(CN)_6_ containing 1.5% PVP were mixed to obtain PVP-protected spheroidal PBNPs [58].

By altering the experimental conditions, Wang and colleagues, in another conceptual study, investigated the mini-emulsion periphery polymerization (MEPP) technique to create PB nanoshells and nanoboxes with tunable sizes [59]. MEPP involves creating mini-emulsion droplets with the use of organometallic surfactants, then allowing the droplets’ periphery for coordination polymerization. In a typical experiment, penta-cyanoferrate-terminated organometallic surfactant poly(ethylene glycol)-b-poly(propylene glycol)-b-poly(ethylene glycol) (EPE-Fe) was employed to generate mini-emulsion droplets with a penta-cyanoferrate perimeter, while water, toluene, and hexadecane were utilized as the solvent. This method helps in the fabrication of spherical nanoshells, crystalline cubic nanoboxes, and tunable-size amorphous solid nanocubes. Later, using a separate non-ferrate co-surfactant in addition to the aforementioned EPE-FE resulted in particles that were either 250 nm or 100 nm in size. The co-surfactant was essential for the fabrication of cubic nanostructures as the pure EPE-Fe has the ability to synthesize only spherical hollow PBNPs. Hollow PBNPs can also be created instead of microemulsions by carefully controlling the chemical (acidic) etching of solid PB nanocrystals [60]. High surface area hematite crystals can be produced by converting PB nanocrystals made with this process into nanoporous Fe oxides through calcinations [61]. The expensive functional predecessors for the MEPP technique prevent scalable preparation, but the precise shell-like microstructures have positive academic implications.

### 2.3. Template Method

Excellent template synthesis of ferritin-protected PBNPs has been undertaken by utilizing a solution containing ferritin-trapped [Fe^III^(CN)_6_]^3−^ by first dissociating apoferritin into its component subunits at pH 2 and then rehabilitating it at pH 8.5 in the presence of [Fe^III^(CN)_6_]^3−^. Small, spherical PBNPs (~5 nm) were formed following the addition of Fe^2+^ to the dialyzed solution. The ferritin cage structure, along with its internal dimensions, played a critical role in determining the size of the PBNPs while also preventing agglomeration. This process facilitated the formation of not only homobimetallic but also heterobimetallic PBNPs. The ferritin-PBNPs absorbed light at 710 nm. In another template-based synthetic protocol using Cowpea chlorotic mottle virus (CCMV) without RNA, monodispersed PBNPs with a diameter of approximately 18 nm were produced through a photo-initiated stepwise reaction. Since the CCMV capsid is assembled and disassembled in a variety of pH solutions, it can encapsulate both [Fe(C_2_O_4_)_3_]^3−^ and [Fe(CN)_6_]^3−^ simultaneously upon light irradiation with a reaction time of less than 12h.

Hollow-structured NPs obtained via chemical etching, template synthesis, and mini-emulsion periphery polymerization (MEPP) technique have high drug loading capacities, strong reactive activities, and enable the creation of multifunctional nanosystems. Cai et al. created hollow, mesoporous gadolinium PB NPs with controllable LSPR, using bovine serum albumin (BSA) as a template. The absorption peak changed to 910 nm when the Gd^3+^ concentration rose and the magnetic resonance imaging (MRI)/photoacoustic imaging (PAI) performance also significantly improved [62].

### 2.4. Hydrothermal Method

Highly efficient photothermal water sterilization using PB nanocages (PBNs) was studied by Jiang et al. [63]. These nanocages were synthesized using a simple hydrothermal method, and possess excellent photothermal properties due to their strong and broad optical absorption in the NIR regions, as well as their efficient photothermal conversion capabilities, which are dependent on concentration and irradiation time. When tested on *E. coli* bacteria, high photothermal bacterial inactivation was observed at approximately 100% upon exposure to NIR radiation for just 5 min, even at low dosages (100 μg/mL). Additionally, when used for sterilization of polluted drinking water containing different bacterial strains, the PBNPs eliminated almost all bacteria upon NIR irradiation. Remarkably, this study also demonstrated that PBNPs could be used to achieve photothermal sterilization even under mild solar irradiation (0.1 W/cm^2^), with a killing efficacy of ~99% upon 10 min of exposure. These findings suggested that photothermal sterilization using solar light has great potential for practical water treatments, and could pave the way for more efficient and sustainable water purification methods. The same research group studied water decontamination with PB-coated ferroferric oxide (Fe_3_O_4_@PB) to combat water-borne diseases [64]. Under solar-light irradiation, the composites photothermally inactivated almost all bacteria in just 15 min by reaching temperatures above 50 °C. Fe_3_O_4_@PB possesses a highly magnetized Fe core that allows magnetic separation and recyclable. This core-shell nano-composite retains its photo sterilization tendency and reusability even after multiple cycles. This research provides evidence of a low-cost, scalable, efficient, and reusable method for improving water quality and safety through photo-sterilization.

## 3. Synthesis of PB Analogues and Ion Doping

Complete substitution (PB analogues or PBAs) or partial substitution (doping) of analogous functional metal atoms into the PB lattice is possible due to the cubic architecture of PBNPs and can further be used to adjust the electrical structure of PB and PBAs. The addition of new functionalities and improvement of the PBNPs can be carried out by easily burning off or leaching out PBAs -CN bridge to create highly porous materials.

The term “PB analogues” describes compounds with a structure resembling that of PB but without the C- or N-bonded Fe atoms but with some similar functional atoms of the general formula AxMy[M’(CN)_6_]z, where A is an alkaline metal cation, and M and M’ are metal cations in oxidation state +2 or +3. For instance, the NPs of gallium PBAs KGa[Fe(CN)_6_]nH_2_O were created by combining the solutions of Ga(NO_3_)_3_ and K_4_[Fe(CN)_6_]. FeCl_2_.4H_2_O and K_3_Co(CN)_6_ were combined to create NPs of the cobalt PBAs. The structural diversity offered by the PBAs broadens the prospective technological and biomedical uses of PB-based NPs and has received enormous attention from researchers. For instance, after adding Gd^3+^ or Mn^2+^, NPs of the gadolinium/manganese PBAs show excellent potential as a contrast agent in MRI. Mn^2+^ is released in response to an acidic environment because the Mn-CN-Fe connection in the manganese PBAs is relatively weak and the absorption is red shifted to 768 nm entering the optically transparent region [65]. The pH-responsive release of Mn^2+^ from manganese PB analogue has been used to track synchronized medication release during chemotherapy for tumours.

Doping is another way to create PBNPs with tunable characteristics. It involves partially replacing Fe atoms with equivalent atoms. The gadolinium-doped PBNPs are effective in MRI due to their exceptional MRI properties. The PBA absorption spectra fall within the visible region with charge transfer bands of similar intensity to pure PBNPs. However, it limits the use of PBAs as PTAs as this region comes within the visible light range. However, recent studies have shown that controlled doping with Mn^2+^ or Zn^2+^ leads to a red shift in CT bands, making them effective photothermal agents (PTAs). Among lanthanide (Er^3+^, Dy^3+^, Tm^3+^ and Yb^3+^) doping, Yb^3+^ exhibits the best results as the maximum absorption wavelength shifts to 816 nm, and photothermal conversion efficiency increases from 46.6% (PB) to 55.0% (YbPB with 9% Fe substituted by Yb^3+^) [36].

## 4. Synthesis of PB Nanocomposites

Nano-chemistry tools have made it possible to enhance the physicochemical properties of PBNPs and their analogues for improved and diversified applications through integration with other materials such as drugs and photosensitizers. A number of PB-based nanocomposites have been developed recently, each with unique physicochemical characteristics, with a particular emphasis on drug delivery, photothermal properties, photodynamic properties, photoluminescence properties, and multimodal contrast agents for multiple imaging techniques. Because they are microporous, their drug-loading capacity remains low. Coating PBNPs with mesoporous organo-silica is a good strategy to improve their surface area and enable them to serve as nano-cargoes for internal delivery. Tian et al. have demonstrated periodic mesoporous organo-silica coating on PBNPs (abbreviated as PB@PMO) which have shown organic-inorganic hybrid frameworks, uniform diameter (125 nm) in addition to high surface area (866 m^2^/g), mesoporous channels (3.2 nm), and high drug loading capacity (260 μg/mg) with exceptional photothermal conversion efficiency [66]. Yue and colleagues created NaNdF_4_@PB core-shell NPs to increase the photothermal conversion efficiency. In comparison to separate NaNdF_4_ and PBNPs, the nanocomplexes have exhibited greater photothermal conversion efficiency thanks to the cross-relaxation pathway between the Nd^3+^ ions ladder-like energy levels and the PBNPs’ continuous energy band. In addition to the photothermal impact, bacteria can be effectively removed by the photodynamic features. Photoresponsive nanocubes, AgPB nanocomposites with an average size of 140 nm and 5–15 nm AgNPs loaded onto them, can rapidly heat up to 50 °C under 808 nm NIR light. This process enhances ROS production and promotes the release of Fe^2+^, Fe^3+^, and Ag^+^ ions, increasing oxidative stress, which significantly suppresses bacterial proliferation within 10 min [67].

Optical imaging represents emerging technology for the diagnosis and treatment of diseases. PBNPs incapability to have luminescent characteristics restricts their usage in optical imaging. A promising strategy is to create nanocomposites consisting of PBNPs with luminescent components. For instance, to enable optical imaging, PBNPs were coupled to biocompatible CuInS_2_-ZnS quantum dots (QDs) with NIR fluorescence emission and carbon dots (CDs) with green photoluminescence emission [68]. Post-synthetic functionalization of 2-aminoanthracene luminophore with K^+^/Ni^2+^/[Cr(CN)_6_]^3−^, PBA NPs produce a bifunctional magneto-luminescent nanosystem. A NaErF_4_ inner core gave multi-layer core-shell nanocomposite inherent photoluminescence capabilities. PB-coated NaErF_4_@NaYF_4_@NaNdF_4_ core/shell/shell nanocrystals showed theranostic capabilities with hyperthermia and switched imaging, encapsulated in a phospholipid PEG micelle (PEG-CSS@PB) under distinct near-infrared (NIR) light activation. The light-to-heat conversion efficiency was ~50.5% of this coated lanthanide-based PB nanoplatform. The utilization of PBNPs in computed tomography (CT) imaging and radiotherapy is constrained by the insufficient X-ray adsorption of PBNPs. The systematic study of the series of AMn^II^[Fe^III^(CN)_6_] (A = K^+^, Rb^+^, Cs^+^) derivatives incorporating Rb^+^ and Cs^+^ ions in the tetrahedral sites of the parent fcc PBA displayed significantly increased X-ray attenuation coefficients. A therapeutic coalescent design was developed based on the strong photothermal effect and T_1_- and T_2_-weighted MR contrast of PBNPs as cores, and radio sensitization and CT enhancement of AuNPs as satellites, named as PB@Au core-satellite nanoparticles (CSNPs) for magnetic resonance (MR)-computed tomography (CT) imaging and synergistic photothermal and radiosensitive therapy. The high X-ray attenuation coefficient of bismuth makes it an excellent candidate for CT imaging and radiation therapy. Chen et al. have affixed BiOCl nanosheets to nanoparticles of the bismuth-based PB analogue to further enhance the X-ray attenuation coefficient for improved CT imaging [69].

## 5. Application in Treatment of Topical Bacterial Infections and Cutaneous Wound Management

### 5.1. Photothermal Antibacterial Effects

Bacterial infections pose a major global health threat due to the ubiquitous nature of bacteria. The excessive and improper misuse of antibiotics has contributed to the rise in antibiotic-resistant bacteria that cause serious risks to public health. The nanomaterial-based remedies offer a promising approach to overcoming incurable bacterial infections by potentially bypassing pathways associated with acquired drug resistance. Due to the efficient charge transfer between Fe^3+^ and Fe^2+^ via the cyanide ion network, nanoscale PB coordination polymers can effectively raise the temperature in a contactless and spatially localized manner by converting NIR light into thermal energy. In comparison to typical photothermal agents (PTAs), the molar extinction coefficient of carbon nanotubes (7.9 × 10^6^ M^−1^ cm^−1^ at 808 nm) [70] and Cu_2_—_x_Se (7.7 × 10^7^ M^−1^ cm^−1^ at 808 nm) [71], PBNPs is significantly greater (1.09 × 10^9^ M^−1^ cm^−1^ at 808 nm), while slightly lower than that of Au nanorods (5.24 × 10^9^ M^−1^ cm^−1^at 808 nm) [72]. Thus, PBNPs demonstrate significant efficiency in converting light to heat. Moreover, the therapeutic success of PBNPs lies in their excellent photostability and dispersibility in both aqueous and biologically relevant settings making them reliable photothermal ablation agents. As reported by Somani et al. *Pseudomonas aeruginosa*, *Staphylococcus aureus*, *Salmonella typhi*, and *Shigella* were the four bacterial strains used to investigate the efficacy of non-nanostructured PB which was found to lack intrinsic antibacterial activity [73].

In 2016, Boukherroub and colleagues tested PVP-coated PBNPs against antibiotic-resistant bacteria using the photothermal effect with the consideration of a therapeutic antibacterial agent [74]. The researchers investigated the PBNPs’ photothermal activity at two distinct near-infrared (NIR) wavelengths: 810 nm and 980 nm. The temperature rise is directly related to the nanomaterial dosage and is greater at 810 nm than at 980 nm. With 10 min of laser exposure at 1 W/cm^2^ irradiance, Boukherroub examined the photothermal antibacterial effect of PVP-coated PBNPs. Both Gram-positive, methicillin-resistant *Staphylococcus aureus* (MRSA) and Gram-negative bacteria, including extended-spectrum β-lactamase (ESBL) and *E. coli*, showed promising results at concentrations lower than 100 μg/mL. The authors also claimed that irradiation at 980 nm exhibited minimal cytotoxicity to the mammalian cells up to a concentration of 50 μg/mL with complete eradication of bacteria. On a similar note, Dacarro et al. created a photo-responsive antibacterial surface and studied the photothermal effect which was seen to be proportional to the absorbance at the irradiation wavelength and was verified with four different lasers at wavelengths of 730, 800, 940, and 1064 nm [75]. Non-toxic cubic PBNPs were anchored on a polyamine-functionalized SiO_2_ glass surface, obtaining a good and homogeneous coverage. Irradiation of these samples with low irradiance of 0.25 W/cm^2^ triggered a microbicidal effect with better therapeutic performance against *E. coli* in comparison to *S. aureus* in an optically transparent region.

One of the recent studies focuses on managing dental plaque caused by carcinogenic bacteria that leads to serious tooth decay among individuals. The synergistic effect of ion discharge and the photothermal response of Ag+ doped PB (AgPB) hydrogel helps in removing multispecies bacterial biofilms and therefore prevents dental caries. The one-pot synthesis of hydrogel (CG-AgPB) upon mixing cationic guar gum (CG) with AgPB under NIR treatment exhibits appreciable in vitro broad-spectrum antibacterial activities. Moreover, the CG-AgPB-mediated hydrogel mitigates the cariogenic bacteria in the rat caries model [76]. Therefore, the biocompatible and injectable hydrogel with ion doping capability holds a promising potential in the therapeutic management of dental plaque. The novel discovery of H_2_S gas-sensitized hyperthermia by Su et al. aimed to display the nano-antibacterial and immunomodulatory activity. The encapsulation of diallyl trisulfide (H_2_S donor) in sealed porous Gd-doped PBNPs leads to overcoming inflammatory damage while fighting bacterial infection. The synthesis of the metal–organic framework (MOF) based PB nanocarrier upon degradation due to the acidic environment of biofilm leads to the generation of diallyl trisulfide that quickly reacted with the overproduced GSH in the biofilms to form H_2_S. The generation of heat flow upon NIR irradiation of Gd-doped PBNPs leads to the eradication of biofilms via H_2_S-mediated gene damage and heat-induced bactericidal action. Moreover, the in vivo experiments revealed the polarization of macrophages that ultimately leads to the release of regeneration-related cytokines. Therefore, this novel strategy assisted in turning the pro-inflammatory milieu caused by infection into a regenerative one leading to the successful promotion of tissue regeneration [77].

Utilization of on-demand photothermally activatable non-toxic PBNPs contained in PVA hydrogel films for thermal bacterial eradication and biofilm mitigation has been reported in the literature[78]. When these PVA-PB films are exposed to NIR light (700 and 800 nm), the local temperature increases swiftly. The increase in temperature reaches a plateau of up to Δ*T* ≅ 78 °C within ≈6–10 s under relatively low laser intensities, *I* ≅ 0.3 W/cm^2^. This localized increase in temperature has a significant impact on the growth of *P. aeruginosa* bacteria. Additionally, the high temperature generated by NIR light irradiation can disrupt the biofilm structure, preventing the bacterial cells from adhering to the surface and inhibiting biofilm growth.

In another study, acetylcysteine-modified PBNPs (AC-PBNPs) have been explored for targeted infection therapy and near-infrared radiation (NIR) photothermal sterilization [79]. In particular, AC-PBNPs are fabricated as a multifunctional therapeutic agent using a co-precipitation method, where PBNPs function as powerful photothermal agents and AC stops bacteria from adhering to tissues and clumping together to form biofilms, reducing mucus secretion and increasing efficacy. Using a 980 nm NIR laser, AC-PBNPs demonstrate potent synergistic photothermal sterilization capabilities in a concentration-dependent manner. While 980 nm radiation is only mildly cytotoxic to mammalian cells, 50 μg/mL of AC-PBNPs kills approximately 74% of Gram-positive *S. aureus* and approximately 75% of Gram-negative *E. coli*, with increased biocompatibility observed in human dermal fibroblast (HDF). Another fascinating feature of this research was observed by subcutaneously injecting AC-PBNPs or PBNP suspension into the epidermis of Balb/c mice. The NIR radiation (980 nm, 2 W/cm^2^) was effectively transformed into local heat to cure a localized *S. aureus* infection in vivo and untreated Balb/c mice were regarded as positive controls. The authors presumed that the bactericidal properties of nanoparticles were responsible for the reduction in scab formation following the injection of AC-PBNPs at the target location. As determined by visual inspection of the infection site and verified by histological examination, the combined effects of AC-PBNP injection and NIR irradiation resulted in commendable wound repair. A high number of inflammatory cells were found to be significantly attenuated in the mice treated with AC-PBNPs and NIR irradiation as compared to untreated but infected mice. As a result, this research may offer a promising method for eliminating bacteria from superficial abscesses on mammalian cells.

Keeping the photothermal characteristics of PBNPs in mind, Wu and colleagues designed a functional photo-responsive hydrogel through the process of free radical polymerization [80]. The electropositive surface of the hydrogels uses electrostatic absorption to tightly encapsulate bacteria. The perturbation of the bacterial membrane’s surface potential prevented bacteria from metabolizing normally, which, in turn, prevented them from respiring. This combined with the synergistic effects of photothermal propensity resulted in the highly effective and quick killing of bacteria even at mild temperatures^.^ The antibacterial potency of hydrogels against *S. aureus* and *E. coli* was found to be as high as 99.97% and 99.93%, respectively. PBNP particles served as cross-linking points due to their interfacial interaction with the chitosan polymer and were also able to strengthen the hydrogel’s mechanical properties. These hydrogels also demonstrated good in vitro biocompatibility with fibroblasts. Furthermore, PBNP-loaded hydrogels demonstrated high effectiveness in in vivo experiments. Compared to the control groups treated with chitosan only and standard gauze, the infected wounds treated with PB-loaded hydrogels healed faster with wound closure observed within twelve days after treatment. Additionally, histological tests showed no inflammation on the PB-treated mice four days after the infection, while all of the test groups still displayed large numbers of inflammatory cells, corroborating the in vitro results.

Li et al. showed that combining low levels of hydrogen peroxide (H_2_O_2_) with hollow mesoporous PB nanoparticles (HMPBNPs) to induce mild hyperthermia has a strong inhibitory effect on bacteria [40]. This resulted in effectively inhibiting the growth of both Gram-positive (*S. aureus*) and Gram-negative (*E. coli*) bacteria and also eliminated *S. aureus* biofilms. In vivo wound healing experiments demonstrated that this antibacterial treatment could be conveniently used for wound disinfection and promote wound healing. In another study, composite photothermal hydrogels having antibacterial activity are created in order to diminish bacterial resistance and reduce injury to normal tissues. Tannic acid and PB were loaded into polyacrylamide hydrogels via precipitation and immersion method with strong light transmittance and adhesion, with the swelling rate reaching ~600% showing improved self-cleaning ability of the nanomaterial. This composite hydrogel demonstrated skin-repairing skills and adjustable and controllable tannic acid release ability through the use of photothermal switches. The addition of tannic acid enhanced the hydrogel’s antibacterial properties and reduced the likelihood of antibiotic resistance. Both in vitro and in vivo experiments confirmed that the hydrogel was biocompatible and highly effective in fighting bacteria, which helped to heal infectious skin defects in Sprague Dawley rats within fourteen days.

Quick and safe sterilization of bacteria-infected wounds, especially without antibiotics, has been demonstrated by using novel photoresponsive MOF heterojunctions that are sensitive to 660 nm light irradiation [81]. The MOF heterojunction shows enhanced photocatalytic performance when exposed to 660 nm red light leading to the generation of ROS. This enhanced photocatalytic performance was combined with the intrinsic photothermal effect of PB after 15 min of 660 nm light irradiation resulting in highly effective sterilization rates of 99.84% and 99.3% against *S. aureus* and its biofilm, respectively. The Fe and zirconium ions released from the PB-PCN-224 composites are found to be biocompatible and non-toxic. Additionally, in vivo studies demonstrate that the PB-PCN-224 MOF heterojunction promotes wound healing in male Wistar rats. After 8 days of therapy, the wounds become noticeably smaller in size in the group given PB-PCN-224 than in the other control groups. The PB-PCN-224 group shows few neutrophils and the majority of the cells are normal, pointing to a minimal infection and validating the potent antibacterial action of PB-PCN244post-8 days of therapy and histological examination of the major organs indicates no structural damage or alteration, ensuring the safe usage of these materials for in vivo applications.

### 5.2. PB with Conventional Antibiotics

The combination of inorganic nanomaterials and organic antibiotics has been proposed as a potential solution for treating bacterial infections. This approach can induce a synergistic action or enhance the efficiency of nanomaterials leading to improved antibacterial activity. The synergistic effect of combining inorganic nanomaterials and organic antibiotics arises from the fact that both materials may target different pathways in bacterial cells. The nanomaterials may act by physically damaging bacterial cell walls, while antibiotics inhibit bacterial growth by targeting specific metabolic pathways. The combination of these two mechanisms may result in a more effective and broader spectrum of antibacterial activity. Alternatively, additional research is needed to elucidate the mechanisms underlying the synergistic effect and to ensure biosafety for potential human applications.

Building multipurpose platforms hastily is important for bacterial point-of-care testing (POCT) and eradication [82]. From this vantage point, vancomycin-doped PBNPs (PB-VANNPs) with high photothermal therapy (PTT) effects have been fabricated for bacterial targeting, dual-function portable sensors, and eradication. Vancomycin is one of the well-known antibiotics on the WHO’s list of necessary medications. In order to generate a complex of PB-VANNPs/*S. aureus*, PB-VANNPs were allowed to interact with Gram-positive bacterial surfaces like *S. aureus*. Attachment of vancomycin to PBNP serves two purposes, viz., (i) it enhances the antibacterial and wound-healing effects, and (ii) serves as a targeting mechanism for bacterial detection. PB-VANNPs have been used to identify Gram-positive bacteria (*S. aureus*) using (i) a portable pressure metre acting as a signal reader, and (ii) a thermal camera serving as a temperature read-out signal for the PB-VANNPs/*S. aureus* sediment with a high degree of sensitivity, with a limit of detection (LOD) of 1.0 CFU/mL. During temperature-based detection, the local temperature elevation can successfully inactivate the bacteria. The antibacterial effectiveness can reach up to 99.8%. In this study, *S. aureus*-infected mice were employed as a model to further illustrate the significant potential of PB-VANNPs as an antibacterial agent. After 5 days of therapy, the wound area in the PB-VANNPs group receiving NIR irradiation was significantly reduced (by 10%), compared to the control groups, according to normalized wound area comparison. According to histological analysis, laser treatment did not result in any noticeable organ morphological abnormalities, demonstrating the extremely low toxicity of PB-VANNPs. The created multifunctional nanoplatform not only offered a simple “mix then test” method for on-the-go highly sensitive bacterial detection, but also made simultaneous, high-efficiency bacterial removal possible.

Surface-enhanced Raman scattering (SERS), which primarily relies on bacteria identification but lacks bactericidal action, is the basis for most of the detection given its ultrahigh sensitivity and fingerprinting potency [83]. With excellent sensitivity and accuracy, Gao et al. created an interference-free SERS platform with a sandwich structure that could simultaneously and consistently identify bacterial colonies in whole blood samples and carry out in situ photothermal elimination. The SERS-active substrate was a worm-like plasmonic gold film (pAu) functionalized with 4-mercaptophenylboronic acid bacteria-capturing units and a 4-mercaptobenzonitrile internal reference probe as it presents a characteristic Raman peak in the Raman- silent region. Vancomycin-modified core-shell PBNPs (Au@PB@Van NPs) were a platform SERS tag. Due to the photothermal synergy of pAu and Au@PB@Van NPs, the antibacterial effectiveness is also quite good. It offers a new technique for in-place sterilization and a new means to detect pathogenic bacterial infections in the early stages of disease with an interference-free Raman mapping strategy in the Raman silent region.

### 5.3. Enzymatic Effects of PBNPs: PBzyme as Antibacterials

PBzymes are nanoscale PBNPs mimicking various enzymatic activities in a natural cascade-like system such as superoxide dismutase (SOD), catalase (CAT), and peroxidase (POD) [84]. These materials possess a highly ordered structure and exhibit unique physicochemical properties that make them ideal for a range of applications in biotechnology, biomedicine, and catalysis. PBzymes are highly efficient at catalyzing a range of reactions, including oxidation, reduction, and hydrolysis, making them valuable tools in many biological and chemical processes. They exhibit high selectivity and specificity, enabling them to carry out targeted reactions with high efficiency and accuracy. Moreover, PBzymes can be easily modified to enhance their catalytic properties, such as by changing the chemical composition or altering their surface structure. Unlike natural enzymes, PBzymes are not susceptible to denaturation, making them highly stable under a range of conditions. This stability allows PBzymes to retain their catalytic activity over an extended period, making them ideal for use in biomedical applications. Interestingly, PBNPs are effective scavengers of ROS because of their capacity to imitate the three antioxidant enzymes mentioned above and contrarily, PBNPs can produce ROS via a Fenton reaction.

**Figure 1:**
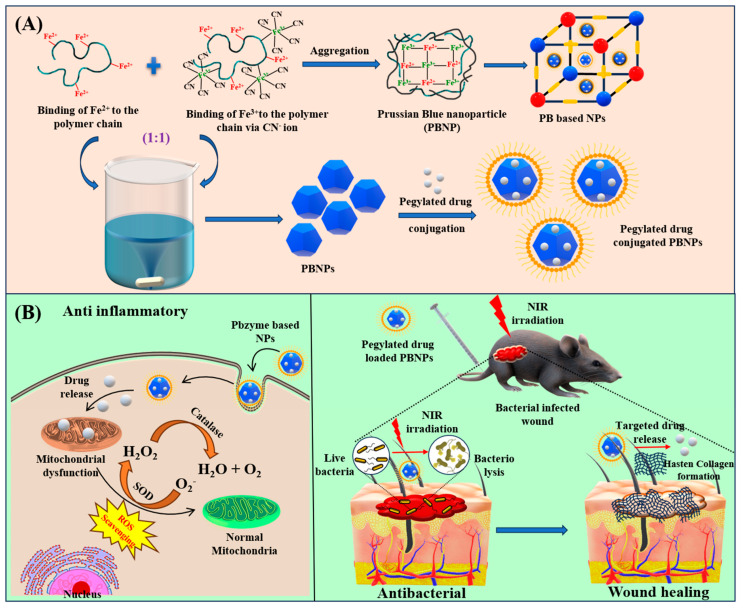
Diagrammatic representation of fabricated Prussian Blue Nanoparticles-mediated treatment of wound healing in the mouse model. (**A**) Fabrication of PBNPs by mixing equimolar concentration of Fe^2+^ and [Fe(CN)_6_]^3−^ via double precursor method. (**B**) Synthesized PBNPs exhibit anti-inflammatory, antibacterial and wound-healing activity. PBNPs showed cytoprotective activity by acting as an excellent ROS scavengers under high oxidative stress thus displaying anti-inflammatory activity. The pegylated drug encapsulated PBNPs facilitated targeted drug delivery when exposed to NIR light that induces the killing of bacteria acting as an excellent antibacterial agent and thus helps in treating infectious wounds.

The main reasons for obstructed wound healing are excessive ROS and untreated inflammation, as they disturb the redox homeostasis and slow down the recovery process. The in vivo therapeutic impact of PBzyme was assessed by Sahu et al. in a skin wound model to modulate inflammation and curb the overproduction of ROS. PB nanozyme displayed H_2_O_2_ degradation capability and at the same time strong superoxide scavenging potential [85]. PB nanozyme demonstrated to be cytoprotective mitigating ROS production under high oxidative stress, in addition to displaying anti-inflammatory activity. The in vivo findings on female ICR mice demonstrated that PBzyme application of 50 μg × 4 groups on 20 × 20 mm^2^ wound once every four days could hasten collagen synthesis, tissue maturation, and wound healing (Figure 1). This finding suggested that the use of several low-dose PB nanozyme treatments (50 μg PB every 4 days) was found to be more efficient than a single 500 μg or 50 μg therapy. Keratinocyte differentiation, neovascularization, and the load of macrophages across the entire wound surface can all be effectively induced by PB nanozyme. Since PBzyme can scavenge ROS and reduce inflammation, the tissue actually demonstrates regeneration properties and the simulated skin wound model heals more quickly. Traditional wound dressings can be infused with nanozyme for more practical or therapeutic uses. This suggests that PBzyme is a reliable therapeutic nanozyme for tissue regeneration and wound healing.

In line with this perspective, Oh et al. fabricated ROS scavenging nanofibers (NFs), composed of chitosan-stabilized PBNPs (PBChi) and poly(vinyl alcohol) (PVA), which imparts significant wound healing and antioxidant properties. PBChi/PVA NFs with low molecular weight of chitosan significantly mitigated ROS production showing better antioxidant activity [86]. These NFs at lower concentrations (10 μg/mL) could appreciably reduce the in vitro ROS level. The NFs owed excellent biocompatibility and showed faster cell proliferation in wound healing assay with a noticeable decrease in size. In a recent study, ultra-small multifunctional copper-doped PB nanozymes (HPP@Cu NZs) have been formulated for the synergistic treatment of infected wounds. These ultra-small nanozymes have been created by modifying hyaluronic acid with polyethylenimine branching, which leads to complexation with copper ions and eventually forms copper-rich PB nanoconstructs. This projected nanomaterial has varied enzymatic performance along with showcasing anti-inflammatory potency. HPP@Cu NZs lead to the slow release of copper ions which lead to the formation of new blood vessels, in addition to bacterial growth inhibition under the influence of 808 nm near-IR laser with removal of unnecessary ROS in microenvironment. The regulation of oxygen levels and anti-inflammation leads to augmented wound repair.

Another study has been conducted to develop a nanozyme–enzyme complex to kill bacteria in a high-glucose environment. Hollow mesoporous PBNPs (HMPBNPs) were loaded with glucose oxidase to synthesize HMPBNPs@GOx catalyzing a cascade reaction of glucose to produce cytotoxic hydroxyl radical in situ at the infectious wound site. The peroxidase-like activity of HMPBNPs@GOx is utilized to facilitate the conversion of H_2_O_2_ into hydroxyl radicals with potential short-range bacterial killing. The therapeutic potential of HMPBNPs is enhanced by GOx-catalyzed oxidation which produces glucuronic acid leading to the creation of an acidic microenvironment. This ensures cascade flow to generate hydroxyl radicals. HMPBNPs@GOx nanocomplex leads to efficient wound healing in in vivo cutaneous wound rat model infected with *S. aureus* [87].

Wang et al. recently reported a MOF- mediated nanozyme synthesized via the pyrolysis action of PB analogues, Mn_3_[Co(CN)_6_]_2_ under air conditions. The fabricated mesoporous nanozyme demonstrated superior CAT-mimicking action that effectively converts H_2_O_2_ into O_2_ within tumour cells, thereby reducing the hypoxic tumour microenvironment. The very efficient photosensitizer loading capacity of nanozymes was made possible by its well-preserved porosity. Additionally, the device demonstrated noticeably improved photodynamic tumour therapeutic efficacy under the NIR laser treatment, both in vitro and in vivo [88].

Peroxidase-like behaviour of PBNPs was explored by our research team in a recent comparative study with and without chitosan coating. The CHPB nanosystem was shown to have the capacity to degrade H_2_O_2_ in order to generate toxic ROS along with exhibition of 635 nm mediated hyperthermia [89]. The antibacterial propensity of the system was investigated both in Gram-positive *S. aureus* and Gram-negative *P. aeruginosa* with significant bacterial killing with chitosan-coated PB NPs with favourable electrostatic interactions at the nanoparticle-bacteria interface. This study emphasized the dual modality of chitosan-coated PBNPs as enzyme-mimicking peroxidase-like activity and hyperthermia with the significance of interactions at the nano-bio interface to help modulate the engineering of PB-based NPs as innovative therapeutic tools in future studies.

The conventional pharmacological interventions remain unresolved for neurodegenerative diseases, such as Parkinson’s disease. Pyroptosis, induced by inflammatory mediators, has emerged as a potential theranostic target for mitigating neurodegenerative disorders. The formation of agonists or antagonists of pyroptosis mediated by inflammasomes has the potential to revolutionize the management of neurodegenerative conditions [90]. However, it is difficult to identify specific compounds that prevent pyroptosis. The utilization of PBzyme as a pyroptosis inhibitor to mitigate the degeneration of neurons in Parkinson’s cell and mice models. PBzyme promotes the shielding of microglia and neurons from MPTP (1-methyl-4-phenyl-1,2,3,6-tetrahydropyridine). PBzyme restores dopaminergic neurons, reduces damage to the mitochondrial membrane potential, and improves motor impairments. Additionally, in a Parkinson’s mouse model generated by MPTP, intracerebroventricular injection of PBzyme decreases dopaminergic degradation and suppresses neuroinflammation. Results from both in vitro and in vivo studies show that PBzyme inhibits the activation of caspase-1 and a multiprotein complex (NLRP3) inflammasomes by scavenging ROS, thus ultimately inhibiting microglia pyroptosis. Overall, the neuroprotective properties of PBzyme as a pyroptosis inhibitor offer insightful mechanistic information as well as a possible therapeutic approach for the management of neurodegenerative disease [91].

### 5.4. PB Analogues (PBAs) as Biocompatible Imaging Agents with PTT Antibacterial Effects

PBNPs and their analogues exhibit exceptional biosafety and biocompatibility properties due to their embellishment with biomolecules and polymers under moderate conditions. In the biomedical sector, PBNPs are employed as therapeutic and diagnostic tools as well as dual magnetic and fluorescent contrast agents in MRI and photoacoustic imaging [92]. The multivariant properties of PBNPs exhibit as an excellent antidote adsorbent for thallium and/or cesium ion poisoning. Moreover, they can also be used as non-antibiotic compounds with antimicrobial properties or as an effective photothermal agent (PAs) in PTT due to their capacity to transform energy into heat [93]. The consequence of different structural characteristics of PB NPs such as shapes, sizes, and charges on their biocompatibility and biosafety assessment were systematically analyzed in vitro and in vivo. Cytotoxicity on cell lines was affected adversely by the positive charge and larger size of PBNPs, while their photothermal conversion and peroxidase activity was enhanced. While in vivo studies showed that PB NPs were well biocompatible with no retention within tissues or organs, however, their bigger dimensions and positive charge hindered their metabolism as well as hepatorenal function [94]. The thorough investigation of biocompatibility and biosafety offers compelling evidence in favour of using PB NPs as an imaging agent or nanodrug carrier.

In 2018, Kim and Huang published the first report on the antibacterial properties, discussing a Ca/Fe(III)-based nanoscale analogue with a PVP coating [95]. These nanoscale analogs (KCa(H_2_O)_2_[Fe^III^(CN)_6_]·H_2_O) possess the ability to penetrate the bacterial cell membrane where they sequester internal Fe through ion exchange to generate “insoluble” PB, preventing bacterial growth. This highly selective and effective mechanism leads to a decrease in Fe concentration. Inhibition of bacterial growth was witnessed over a period of 9 h with 0.8 logarithmic units of viable cells remaining in a dose-dependent manner.

Interestingly, the metal-doped PB displays a red shift in absorption spectrum which increases the photothermal efficiency of nanomaterials when exposed to NIR radiation. A similar pattern of behaviour was seen with Zn-doped PB as well. As an exogenous antibacterial antibiotic, Li et al. created zinc-doped PB (ZnPB) in 2019 [96]. By freeze-drying, ZnPB nanocubes with varying levels of doping were created, and the maximum absorption was observed at 740 nm, enhancing their photothermal ability at 808 nm. It demonstrated the broad-spectrum bactericidal effect of ZnPB-3, with the highest level of Zn doping. ZnPB has been shown to inhibit methicillin-resistant *S. aureus* (MRSA) in animal wound models by promoting infiltration and release of Zn^2+^ ions into bacteria under photothermal effects. This kills bacteria by altering cellular metabolic pathways without causing systemic toxicity. It was discovered that ZnPB-3’s combination of ion release and local PTT enabled the quick clearance of MRSA from the wound surface. They also evaluated the antibacterial effectiveness and toxicity to healthy tissues at similar temperatures. The findings indicate that after 15 min, the bacteriostasis rate exceeded 90% when the PTT’s highest temperature was above 55 °C. However, the neutrophil-mediated inflammatory responses were observed after 15 min when the PTT temperature exceeded 60 °C. By the activation of tissue re-modelling genes, low laser flux and high photothermal conversion efficiency, ZnPB enhance collagen deposition and aids in wound repair. A wide range of antibacterial treatments are made possible by the interaction of ion release and local PTT effects which clarifies the antibacterial application.

The performance of PBNPs PTT is significantly influenced by the electron density of its cyanide bonds. Recent studies, however, have pointed out that adding a PB shell to the core of lanthanide nanomaterials could significantly improve its photothermal efficiency [36]. Doping of PB nanocubes with a range of d-block elements, such as ytterbium PB-Yb exhibited a significant PTT effect at 808 nm as the absorbance maxima were red shifted to 816 nm. Structural analysis results indicate that the replacement of 9% of the Fe (Fe^2+^ or Fe^3+^) by Yb^3+^ in the PB-Yb results in an increase in the electron density of cyanide bonds and enhanced PTT performance, as evidenced by increasing the photothermal conversion efficiency from 46.6% (PB) to 55.0% (PB-Yb). Thus, doping of lanthanide ions into PB was a quick and easy technique to increase its PTT effects and proved to inhibit *E. coli* proliferation under 808 nm laser irradiation.

A triple-layered photothermal nanocomposite comprising copper sulphide (CuS), gold (Au), and ZnPBA (CuS@Au@ZnPBA) for drug-free therapy for bacterial wound infection is devised. On exposure to an 808 nm laser, CuS@Au@ZnPBA effectively generates heat thus, eliminating bacterial infections and promoting the growth of collagen. This is because CuS@Au@ZnPBA releases Zn^2+^ ions that can upregulate genes associated with collagen deposition and also kill bacteria in synergism with the PTT effect [97].

### 5.5. Silver Containing PB in Combating Microbial Resistance

Since time immemorial, silver, silver ions and nanometric silver have been extensively utilized for their antimicrobial potential. However, silver-mediated toxicity limits their utilization and clinical translation. The combination of FDA-approved PB or PBA with silver either as an analogue, dopant or in a composite formulation is hypothesized to have antibacterial capabilities. To our knowledge, the first nanocomposite combining silver and PBA was reported in a study conducted by Mukherjee and Patra, in which the core-shell nickel-prussianblue@silver nanocomposites (NiPB@AgNC) provided stability and biocompatibility assessed by in vitro studies and also by ex vivo chicken embryonic angiogenesis assay [98]. NiPB@AgNC demonstrated high stability in a 10% saline solution at physiological pH, representing an improvement over bare AgNPs, which are typically sensitive to high ionic strengths, particularly in the presence of chlorides. This increased stability may be attributed to the double-layer NiPB exterior coating, which prevents AgNPs from dissolving rapidly and reduces the formation of AgCl in saline solutions. This nanomaterial exhibits significant antibacterial properties in a dose-dependent manner by measuring zone of inhibition and growth inhibition studies as compared to traditional antibiotics (penicillin and streptomycin) for *E. coli* and *B. subtilis*. The antibacterial mechanism is attributed to the slow and sustained release of Ag^+^ for 48 h due to the double-layer coating of NiPB, thus, confirming the beneficial impact of NiPB shell.

Patra and colleagues synthesized a PVP stabilized silver-PBA NPs (Ag_3_[Fe(CN)_6_]; SPBANPs) by complete replacement of ferrous ions with silver ions, resulting in a coordination polymer that exhibited maximum absorption at 400 nm, which is unsuitable for potential in vivo irradiation. The nanomaterial demonstrated promising antibacterial activity against *E. coli*, *P. aeruginosa*, *K. pneumoniae*, and *B. subtilis* comparable to traditional antibiotics like streptomycin, penicillin, kanamycin, and gentamicin. Its mechanism aligns with typical silver actions, including increased lipid peroxidation, membrane damage, and reduced expression of catalase and superoxide dismutase [37].

In a study addressing the urgent need for therapeutics to treat diabetic wounds infected by drug-resistant bacteria, nanocomplexes known as PB@PDA@Ag were developed to eradicate multidrug-resistant bacteria and accelerate wound healing in a MRSA-infected diabetic model, aided by laser irradiation [38]. PBNPs of 90 nm were coated with polydopamine (PDA), which was further functionalized with AgNPs after interacting with the PDA surface followed by in situ reduction forming ~10 nm spherical AgNPs onto the PB@PDA surface. The presence of AgNPs onto the surface did not interfere with the photothermal capability of the PBNPs, instead provided an inherent antibacterial property to the nanocomposite. *E. coli*, *S. aureus*, MRSA and ampicillin-resistant *E. coli*, were tested against PB@PDA@Ag leading to a clearance of >99% cells when this hybrid material was irradiated with 808 nm laser. The evaluation of mechanism ascertained exceptional oxidative stress and hampered ATP production (declining to 96.7%). Glutathione oxidation due to the release of Ag^+^ ions were augmented under irradiation synergy. In addition, in vivo study on female Balb/c mice and subsequent data analysis confirmed the wound healing property of PB@PDA@Ag in MRSA-infected diabetic skin wounds. This is achieved by increased VEGF (vascular endothelial growth factor) expression that promotes the generation of new blood vessels.

Along a similar line of research, our group fabricated silver-doped PB. This citrate-stabilized nanoframework had a 10% silver content with respect to Fe concentration. The partial substitution of Fe centres of PB by other metal ions causes a red shift in the absorption maxima. This property facilitated the assessment of the inherent photothermal effect and antibacterial property under 635 nm laser irradiation. For the same silver concentration, this Ag-doped PB nanosytem produced same intrinsic antibacterial property against *P. aeruginosa* and *S. aureus*, while undoped PB, as expected showed negligible intrinsic effect. The Ag-doped PB nanoframework leads to the complete removal of viable bacterial cells when irradiated by a 635 nm red laser leading to the triggering of a photothermal effect for enhanced antibacterial propensity [99].

## 6. Limitations and Challenges of PBNP Applications

While PBNPs exhibit great potential across various biomedical sectors, several limitations and challenges hinder their broader use. The major concerns of PBNPs are the biocompatibility and potential toxicity.

### 6.1. Long-Term PB and PBA Toxicity

The non-toxicity of PBNPs is attributed to the strong ability of the cyano group of Fe^2+^⋯C≡N⋯Fe^3+^ sequence to bind to Fe ions, preventing them from producing toxic to healthy cells [10]. Although PBNPs are generally regarded as safe, their long-term toxicity, bioaccumulation, and effects on different organs remain insufficiently explored, especially in clinical settings. Prolonged exposure or high doses may lead to unforeseen side effects, which necessitates thorough in vivo studies to establish comprehensive safety profiles. Thus, metabolic long-term fate both in vitro and in vivo along with pharmacokinetics profiles and possible toxicological behaviour of PBNPs and its analogues in animal models should be systematically evaluated to satisfy regulators before clinical trial. The routes of administration for pharmaceutical formulations containing PBNPs or their derivatives, considering the intrinsic toxicity, generally include systemic and topical applications. The subcutaneous, intravenous and topical routes minimize toxicity risks while maximizing therapeutic benefits. The subcutaneous or intravenous application of NPs provides direct routes for systemic distribution for quick therapeutic response with minimum toxicity, depending on the dose optimization. On the contrary, the topical application involves localized delivery with reduced systemic exposure and minimum toxicity [100].

The comprehensive toxicological assessments of PBNPs pose challenges for their use in clinical sectors. Toxicity studies indicate that intravenous injection of PBNPs at a low dose of 5 or 10 mg/kg did not result in significant toxicity in mice. However, mice administered a higher dose of 20 mg/kg showed signs of toxicity, including a reduction in appetite and weight loss. Further, in one of the studies, the in vivo toxicity of PDIM NPs was tested and the results revealed the potential safety and biocompatibility of structured nanomaterial at different doses making them promising antibacterial agents. Along a similar line of research, Wu and colleagues synthesized drug-loaded biomimetic PBNPs (PB@CS-5@M) that significantly outline no toxic effects on major organs when administered intravenously in mice thus, the nanostructured material exhibits outstanding biocompatibility without toxic side effects, making it suitable for advancing to in vivo clinical trials [101].

The presence of cyanide groups in PBNPs will cause long-term potential toxicity [102]. Therefore, synthetic strategies to produce biodegradable, ultra-small PBNPs for rapid metabolism and degradation in the body are crucial [103]. For various biomedical applications, PBNPS and PBA NPs require circulation, extravasation, and accumulation at specific sites. Thus, appropriate surface modification and functionalization with citric acid [104] and PEG [105] can stabilize these nanomaterials with better physiological stability, prolong the blood circulation time, improve the biocompatibility, and reduce other toxicity to the normal tissues [106]. Furthermore, the toxicological profiles of major organs largely depended on the composition, particle morphology, size attenuation, surface modification, and physiological protein absorption gradient [107]. Even though PBNPs are a nontoxic and FDA-approved compound, a thorough investigation of biodistribution, pharmacokinetics, and long-term toxicity is essential to provide a reference for their clinical application.

### 6.2. Scalability of PB NPs

Another challenge lies in the scalability of PBNP synthesis. Achieving precise control over particle size, shape, and surface modification is essential for their therapeutic efficacy, yet it can be difficult to maintain consistency in large-scale production, limiting their further application from laboratory to industrialization. Additionally, PBNPs’ stability in biological environments, particularly in the presence of proteins and enzymes, can impact their efficacy, as they may undergo premature degradation or aggregation.

### 6.3. Combination of Therapies on PB-Based Nanoplatforms

Furthermore, there are limitations in understanding the full extent of their pharmacokinetics, biodistribution, and clearance mechanisms. Since PBNPs are relatively new in nanomedicine, more research is needed to optimize their delivery, targeting efficiency, and interactions with biological systems. Lastly, while PBNPs have demonstrated wound healing, anti-inflammatory and antibacterial properties, integrating them into existing treatment protocols, such as combining them with antibiotics or other therapeutics, requires careful consideration to avoid potential drug resistance or adverse effects.

In conclusion, despite these challenges, the development of PBNPs holds immense potential in nanomedicine, such as in the treatment of diseases caused by ROS as PBNPs can catalytically convert ROS into O_2_ or H_2_O [108]. Addressing these limitations through advanced research and technological innovations will be crucial to unlocking their full therapeutic potential.

## 7. Conclusions and Future Perspective

PB is an ancient pigment that has gained tremendous popularity in nanomedicine due to its nanoscale properties. The exploration of PBNPs for theranostic is a current area of interest with wide applications in various biomedical applications. Recently, there has been a growing demand for using PBNPs as antibacterial agents. This review has comprehensively described the synthetic methodologies and structural tuning of PBNPs as a multimodal nanoplatform to pose as antimicrobial agents. The concept of using PB to kill bacteria arose from its absorption spectrum, which falls in the visible-NIR region, similar to photothermal therapy in cancer. Interestingly, the multiple oxidation states of PB nanozymes have accelerated the exploitation of this nanoscale material to exhibit multicatalytic properties. PBAs have been shown to scavenge excessive ROS and decompose H_2_O_2_ in the system’s microenvironment, generating oxygen to alleviate hypoxic conditions. Simultaneously, they produce ROS, to combat specific diseases. Additionally, various novel design strategies have led to a plethora of possibilities such as an inherent antibacterial property that can be developed when PB is functionalized with organic molecules or antibacterial ions. These two effects can synergistically act to enhance sustained intrinsic impact in materials which can also be improved by NIR light irradiation. However, comparing the effectiveness of these nanostructures is difficult due to the various assays and different conditions and concentrations used to evaluate antibiofilm and antibacterial effects. Typically, concentrations of PB and its derivatives used in studies reviewed range from 5 to 200 µg/mL for suspension and even lower for films and monolayers at an interfacial junction. Studies on both Gram-negative and Gram-positive bacteria exhibited good performance with different susceptibilities depending on the structural modifications of PB/PBA NPs. In some studies, PBNPs or their analogues have been tested against ampicillin-resistant MRSA and *E. coli*, with no cases of resistance observed so far. Furthermore, the capacity of PB/PBA NPs to participate in the restoration of skin tissue by boosting blood flow, angiogenesis, and collagen deposition during tissue repair, makes them a potential theranostic in future developments.

The recognized biocompatibility and versatility of PB offer numerous possibilities in the orientation toward the diversified design of PB-based nanoparticles, which are currently under active development. Whether through complete substitution (PB analogue), partial substitution (doping), and/or antibiotic loading, PB/PBA NPs have emerged as pioneering nanodrug carriers with excellent cellular penetration, minimal cytotoxicity, and notable chemical stability. The structural control of PB/PBA NPs further enhances their specific surface area, increasing antibiotic loading capacity. Based on the recent literature reviewed in this article, we anticipate significant advancements and growing interest in this field over the next few years.

From this perspective, the objective of this compilation centres on the utilization of this fascinating nanoplatform, PBNPs or PBAs-based nanoparticles to combat bacterial infection, which could pose a serious global health threat in the form of a ‘silent pandemic’ in the near future.

## Figures and Tables

**Table 1:** Summary of Synthetic Strategies for PBNPs.

S. No.	Modified PBNPs	Synthetic Strategies	Composition	Shape	References
1	**PB analogues** Fe4[Fe(CN)_6_]_3_	Chemical coprecipitation	Poly(vinylpyrrolidone) (PVP) and K_3_[Fe(CN)_6_]·_3_H_2_O	Nanocubes	[16,17,18,19]
	Mesostructured Zn_3_[Fe(CN)5NH_3_]_2_·_3_H_2_O	Ligand-assisted template	octadecylpyrazinium bromide Na_3_[Fe(CN)_5_NH_3_]·_3_H_2_O, and Zn(NO_3_)_2_ (linking agent)	Hexagonal mesostructured framework	[20]
	Monocrystalline Co_3_[Fe(CN)_6_]_2_·1_2_H_2_O	Controlled chemical etching	Cobalt chloride, sodium citrate, PVP, and K_3_[Fe(CN)_6_]	Solid and Hollow nanocubes	[21]
	Co_3_[Co(CN)_6_]_2_	Microemulsion	K_3_[Co(CN)_6_], n-pentanol, cyclohexane and CTAB	Cubes and rods, polyhedral truncated nanocubes	[22]
	Multimetal CoNi-HCF@Ni-HCF	Coprecipitation	CoCl_2_, NiCl_2_, sodium citrate, Na_4_Fe(CN)_6_, and PVP	Nanocubes	[23]
2	**PB microcrystals** Fe4[Fe(CN)_6_]_3_	Selective chemical etching and thermal decomposition	K_4_[Fe(CN)_6_] and Cetyltrimethylammonium bromide (CTAB)	Elongated nanocubes	[24]
3	**PBNPs and Nanocrystal superlattices** FeII_3_[FeIII(CN)_6_]_2_	Reverse microemulsions	Ammonium iron(iii) oxalate (NH_4_)_3_ [Fe(C_2_O_4_)_3_], and ammonium ferricyanide solutions (NH_4_)_3_[Fe(CN)_6_]	Square (cubic) superlattices	[11]
4	**Chitosan-PB nanocomposites** K_2_FeII[FeII(CN)_6_]	Coprecipitation	K_3_Fe(CN)_6_, and chitosan (protective matrix)	Spherical NPs	[23]
5	**Nanoporous metal oxides** FeII_3_[CoIII(CN)_6_]_2_KCoII4 [FeIII(CN)_6_]_3_	Coprecipitation	FeCl_2_⋅_4_ H_2_O, trisodium citrate dihydrate, and K_3_[Co(CN)_6_]	Nanocubes	[25]
6	**Nanoporous Mn-based PBNPs** Mn_2_[Ru(CN)_6_].xH_2_OMn_3_[Co(CN)_6_]_2_.xH_2_OMn_3_[Mn(CN)_6_]_2_·xH_2_O Cu(H_2_O)_2_[Pt(CN)4]·4H_2_O	Coprecipitation	Trisodium citrate dihydrate (TSCD), manganese (II) acetate aqueous solution, K_4_[Ru(CN)_6_], K_3_[Co(CN)_6_], and K_3_[Mn(CN)_6_]	Nanocubes	[26]
7	**Nanoporous Nickel Oxide PBNPs** Ni(H_2_O)_2_[Ni(CN)_4_]·_4_H_2_O	Coprecipitation	NiCl_2_⋅_6_ H_2_O, TSCD, and K_2_[Ni(CN)_4_]⋅x H_2_O	Nanoflakes	[27]
8	**Cyano-Bridged Cu–Pt PBNPs** Cu(H_2_O)_2_[Pt(CN)_4_]·_4_H_2_O	Coprecipitation	copper(II) acetate, trisodium citrate, and potassium tetracyanoplatinate(II)[(K_2_Pt(CN)_4_]	Nanoflakes	[28]
9	**PB solid nanospheres with multi-shells** PdCo/Pd-Co(CN)_6_]x·nH_2_O	Selective chemical etching	PVP, and K_3_[Fe(CN)_6_]	Solid Nanocubes	[29]
10	**PB nanoframes** FeHCFe	Hydrothermal	PB Nanocubes and benzoic acid	Nanoframes	[30]
11	**Potassium manganese hexacyanoferrate superstructures** K_2_Mn[Fe(CN)_6_]	Coprecipitation	Manganese(II) acetate tetrahydrate (MnAc_2_·_4_H_2_O), K_2_EDTA·_2_H_2_O, PVP and K_3_Fe(CN)_6_	Octahedra	[31]
12	**ClO4-doped polypyrrole-coated nanocomposite** NMHFC@PPy	Coprecipitation	Manganese acetate, Na_4_Fe(CN)_6_ 10H_2_O, dimethyl carbonate, and sodium citrate	Irregular NPs	[32]
13	**Carbon cloth PB analogue@polyaniline** C@PAn@CoHCF	Coprecipitation	Carbon cloth (CC), (NH_4_)_2_S_2_O_8_, trisodium citrate, CoCl_2_, Na_4_Fe(CN)_6_	Cubes	[33]
14	**Crystallized PBNPs** Na_2_Fe_4_[Fe(CN)_6_]_3_	Hydrothermal	Fe^3+^ and [Fe(CN)_6_]^4−^	Microsphere	[34]
15	**Zinc hexacyanoferrate** Zn_3_[Fe(CN)_6_]_2_	Coprecipitation	ZnSO_4_, Na_2_C_2_O_4_, and K_3_[Fe(CN)_6_]	Nanocubes	[35]

**Table 2:** PB-based Nanoplatforms as Antibacterial Agents.

S. No	Nanoscale Modifications of PBNPs	Target Bacteria	Routes of Administration of PBNPs and Its Derivatives	Mechanism of Action	Results	References
1	Fabrication of Ytterbium—doped PBNPs (PB-Yb)	*E. coli*	NIR irradiation of bacterial colonies containing nanostructured PBNPs	Stimulates photothermal mediated disruption of bacterial membrane integrity due to ROS generation	Enhanced photothermal effect leads to better bactericidal property	[36]
2	Silver hexacyanoferrate PBNPs stabilized with poly(N-vinyl-2-pyrrolidone) (SPBANPs)	*E. coli*, *K. pneumonia*, *P. aeruginosa and B. subtilis*	Intraperitoneal injections	Generation of ROS due to release of silver ions leading to loss of membrane integrity	Enhanced antibacterial efficacy	[37]
3	Functionalization of polydopamine with silver NPs on the PBNPs surface (PB@PDA@Ag)	*E. coli*, *S. aureus*	Topical administration followed by NIR (808 nm)	Synergistic production of ROS, mitigating ATP production and interference with bacterial metabolism	NIR-assisted PB analogue enhanced bactericidal efficiency in a sorter interval	[38]
4	PBNPs doped with chitosan and gelatin-packing film (CS/Gel/PB)	*S. aureus*, *E. coli*	Direct mixing of the cancer cell line with nanostructured PBNPs	The electrostatic interaction between the PBNPs films and bacterial cell membrane disrupts the membrane’s potential, causing leakage of cellular contents, ultimately leading to bacterial death	Enhanced antioxidant, thermal, mechanical and water resistance properties, increased adhesion to bacteria leading to sustained antibacterial activity	[39]
5	Association of hydrogen peroxide on hollow mesoporous PBNPs (HMPBNPs)	*S. aureus*, *MRSA*	Topical administration	Photothermal-assisted bacterial destruction	Effective bacterial eradication and elimination of biofilm via mild hyperthermia induced by HMPBNPs	[40]

**Table 3:** Summary of PB-based Nanoplatforms in Anti-inflammatory, Wound Healing Potential, and Other Associated Diseases.

S. No	Nanoscale Modifications of PBNPs	Routes of Administration of PBNPs and Its Derivatives	Application	Mechanism of Action	Outcome	References
1	Surface coating of CpG oligodeoxynucleotide on PBNPs (CpG-PBNPs)	Intratumoral administration	Nanoimmunotherapy	Enhanced the adjuvanticity and antigenicity of treated tumours, effectively generating immunological memory	Persistent regression of tumour and concurrent adjuvant, antigenic and cytotoxic effects to long-term immunological response	[41]
2	Surface functionalization with polydopamine on hollow mesoporous PBNPs loaded with curcumin (HMPB@Cur@PDA)	Subgingival administration	Treatment of dual responsive maxillofacial infection by acting as anti-inflammatory and ROS regulator	Act as a ROS scavenger and anti-inflammatory agent, and stimulates macrophage polarization	Sustained drug release, decreased inflammatory response, and enhanced tissue recovery	[42]
3	Modified copper-hyaluronic acid PB nanozymes (HPP@Cu NZs)	Topical administration	Anti-inflammatory and wound healing	Exhibits NIR-assisted bactericidal properties by acting as ROS scavenger as well as promotes cell proliferation and migration	Exhibits faster vascularization, epithelialization, and collagen deposition	[43]
4	Functionalized PBNPs with polydopamine and curcumin (HMPB@Cur@PDA)	Subgingival administration	Maxillofacial infection	Modulates immune response by synergistically regulating inflammatory microenvironment and scavenging ROS	Lowered periodontal inflammatory response and improved tissue repair	[42]
5	Nano formulation of PBNPs hydrogel modified with growth factors	Systemic administration	Wound healing	Mitigate oxidative stress effectively by modulating the immune microenvironment and promoting angiogenesis and tissue repair	Accelerates angiogenic efficacy of VEGF, promotes wound closure, and reduces scarring	[44]

## Data Availability

The study did not report any new results or data.
